# Development and Implementation of a Culturally Appropriate Education Program to Increase Cervical Cancer Screening among Maasai Women in Rural Tanzania

**DOI:** 10.5334/aogh.2503

**Published:** 2019-10-17

**Authors:** A. Lidofsky, A. Miller, J. Jorgensen, A. Tajik, K. Tendeu, D. Pius, E. Mallange, A. Dougherty

**Affiliations:** 1 Robert Larner College of Medicine at the University of Vermont, Burlington, VT, US; 2 University of Michigan Department of Emergency Medicine, Ann Arbor, MI, US; 3 Kaiser Permanente-Santa Rosa, Santa Rosa, CA, US; 4 Brown University/Rhode Island Hospital, Providence, RI, US; 5 Wasso Designated District Hospital, Ngorongoro, TZ; 6 Department of Obstetrics, Gynecology and Reproductive Sciences at the Robert Larner College of Medicine at the University of Vermont, Burlington, VT, US

## Abstract

**Background:**

In Tanzania, the incidence of cervical cancer is nearly ten times that found in the US. Tanzanian women of the traditional Maasai tribe are financially and educationally marginalized and face a language barrier that reduces access to health care. While cervical cancer (CACX) screening programs are available locally, in our experience, Maasai women were less likely to use these services compared to local women of other tribal backgrounds.

**Objectives:**

A novel patient education program was designed to teach Maasai women about the natural history of cervical cancer and available screening and treatment. The program addressed the importance of preventative health and informed consent. Additionally, we sought to better understand the specific barriers Maasai women face in accessing and utilizing CACX screening services.

**Methods:**

The program used simple, scripted language translated into Maa language, the Maasai native language, with accompanying culturally appropriate 3D models. The effectiveness of the program was evaluated through pre and post-intervention surveys administered to Maasai and non-Maasai women as well as local healthcare providers, assessing knowledge of cervical cancer, screening, and treatment. Paired t-test analyses were used to analyze significance. Extensive question and answer sessions followed the education sessions from which additional barriers to screening were identified.

**Findings:**

Maasai women had minimal understanding of preventative health services prior to the intervention. While all groups showed an increase in knowledge following the education program, Maasai women demonstrated the greatest statistically significant improvement in knowledge. The proportion of Maasai women in attendance to CACX screening clinics increased by 18% after the intervention.

**Conclusions:**

Through a culturally sensitive and accessible patient education program, Maasai women gained knowledge of cervical cancer screening and treatment. This program serves as an adaptable model for other marginalized populations to increase patient understanding and informed consent, and to address issues that pertain to underutilization of health care services.

## Introduction

Carcinoma of the cervix (CACX) poses significant morbidity and mortality worldwide and is the leading cause of cancer-related death in women in sub-Saharan Africa [1]. Nearly all cases of CACX are attributed to human papillomavirus, the most common viral infection of the reproductive tract. In 2012, 84% of the new cases of CACX worldwide were estimated to affect women living in low-income regions [2]. Early presentations of CACX are generally asymptomatic and treatable. If symptoms present, in most cases, the cancer is at an advanced stage and is not curable. In low-resource settings, treatment options for advanced CACX are limited [2]. CACX is a fitting disease model for screening interventions, given its lengthy natural history with a long asymptomatic period. Indeed, the annual incidence of cervical cancer decreased by 80% after routine screening with Papanicolaou (Pap) testing was adopted in the US [3]. However, routine Pap testing is not feasible in resource-limited settings due to high cost and the need for cytology services and trained personnel [3]. In 2011, it was estimated that there were only 15 pathologists in the entire country of Tanzania, or one per 2.5 million people [4].

Beyond the conventional Pap test or the more commonly used liquid-based cytology, other methods of CACX screening include visual inspection with acetic acid (VIA), and HPV testing for high-risk HPV types [2]. VIA is recommended by the World Health Organization as an alternative screening modality as it does not rely on specialized infrastructure or delayed interpretation of results. The components required are readily available in resource-limited areas and women can be offered immediate treatment in a single visit (often called the see-and-treat approach). In VIA, the cervix is washed with acetic acid which allows for identification of abnormal cells and precancerous lesions. If lesions are identified as appropriate for treatment, immediate cryotherapy may be offered [2].

Despite the affordability, efficiency, and efficacy of the VIA intervention to screen CACX in low-resource settings, many Tanzanian women do not have access to routine screening because of the geographic barriers of living in a rural environment, such as lack of transport and considerable distances to health care centers. In 2014, a team from the University of Vermont Larner College of Medicine (UVM LCOM) partnered with the Wasso District Hospital in Ngorongoro District, Tanzania, and subsequently launched the district’s first CACX screening clinic in 2016. In 2017, the clinic became mobile and reached several remote villages in the region thereby addressing issues with access. However, data from the mobile clinic showed that although women were utilizing the services, Maasai women were not proportionally represented in the screened population.

The Maasai tribe makes up a small subset of over 100 ethnic groups in Tanzania. Unique to the Maasai and only a handful of these groups is their pastoral nature. Members originally inhabited a vast region stretching from the Indian Ocean to Lake Victoria. In the late 19^th^ century, British colonization took over their population, and restricted their land to a region that spans from southern Kenya to northern Tanzania in the Rift Valley [5]. This is the present day Ngorongoro district, where the Maasai comprise a majority of the population [6]. Maasai people have been isolated from mainstream society given these geographic constraints. The Maasai all speak the same language, Maa, but are divided into 12 sections, or *oroloshon*, which are distinguished by different political and cultural customs [7]. Among all sections, the Maasai tribe is deeply grounded in their patriarchal culture, which often keeps female children out of school and prevents them from learning the national language, KiSwahili [5,8]. Language barriers have limited opportunities for Maasai individuals to seek employment and to interact with developmental tourism, NGOs, and western health groups. Most medical providers are KiSwahili speakers and do not speak Maa. Non-Maasai cultures generally do not share perspectives of the Maasai surrounding health, illness, disease prevention, and treatment [7]. For example, it is common among Maasai families to seek healthcare only once very ill, as it is considered culturally taboo for someone to be labeled as sick. These factors contribute to poor healthcare outcomes and lack of utilization of preventative healthcare services [9].

Over the last two decades, culturally sensitive patient education has emerged as a theme in patient-centered care. Cultural sensitivity has been defined in the literature as “the ability to be appropriately responsive to the attitudes, feelings, or circumstances of groups of people that share a common and distinctive racial, national, religious, linguistic, or cultural heritage [10].” By understanding attitudes, feelings, and circumstances of a group of people, and crafting education specific to that cultural heritage, the willingness of a patient to accept treatment increases [11,12,13]. A double-blinded, randomized control trial found that patients with low levels of society immersion subjectively perceived culture-sensitive patient information material to be superior compared with standard translated material [14]. Additionally, culturally sensitive education efforts have been shown to positively affect patient-related, professional, and organizational outcomes. A meta-analysis reported that five studies showed significant improvements on patient-related outcomes after receiving culturally sensitive care. These outcomes included improving diabetic control, lowering hemoglobin A1c levels, and weight loss. Patient satisfaction and a mutual understanding between the patient and the clinician also improved [15].

Given this, we designed a culturally sensitive education program using evidence-based methods to minimize language and cultural barriers for Maasai women. Our objective was to educate Maasai women on cervical cancer, available screening, and early treatment options, in order to increase utilization of CACX screening services and ensure informed consent for this marginalized population.

## Program Description

A culturally sensitive educational program was implemented in a pre-existing partnership between the University of Vermont Larner College of Medicine and the Wasso District Designated Hospital in Ngorongoro, Tanzania, which was started in 2014. Through joint priority setting, the partnership agreed to start a CACX “screen and treat” program using VIA and cryotherapy. Initially, Tanzanian providers were trained in VIA and cryotherapy. In 2017, with philanthropic funding, the necessary infrastructure to continue the program independently was purchased. This included gas tanks and a cryotherapy device. CACX screening was available in three locales. Demographic data was collected, and subsequent analysis showed 35% of women attending the 2017 clinic had no form of schooling, 36% had some primary, and only 29% had some secondary school education. Forty-eight percent of all women attending the screening clinics were Maasai, despite comprising a majority of the population in that region [6].

**Figure 1:**
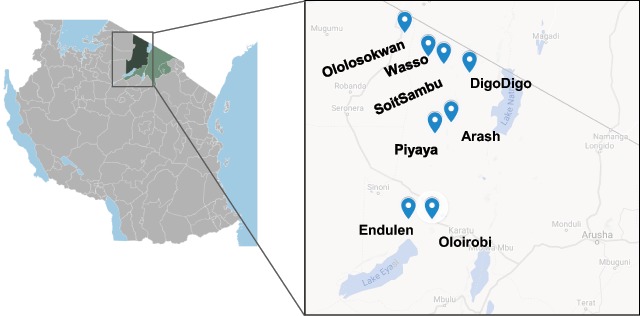
**Clinical Site Locations.** Highlighted in dark green is the Ngorongoro district of the Arusha province of Tanzania (the remainder of which is shaded in light green). This figure was made using Google Custom Maps and an image from Wikipedia (ZH – Own work, CC BY-SA 3.0. https://commons.wikimedia.org/w/index.php?curid=15893018).

By 2018, in cooperation with the Tanzanian Ministry of Health, a full-scale cervical cancer prevention outreach program was launched. The program offered HPV vaccination, screening for cervical pre-cancer, and treatment with VIA and cryotherapy. The clinic, designed to be transportable provided that a roofed structure was available, allowed women residing in remote enclaves, for whom transport was difficult, to access these critical services. Clinics were available to women over the course of six weeks in eight locations across the district (Figure 1).

Given prior experiences, with limited numbers of Maasai women availing themselves of CACX screening despite making up most of the population, a formalized, culturally sensitive CACX education program was designed and implemented in 2018.

## Methods

An orally presented educational program was developed that simplified abstract medical concepts using language offered in KiSwahili (national language) and Maa (Maasai tribal language) at a maximum fourth grade reading level. The presentation was accompanied by three-dimensional models and in situ anatomical depictions to aid in the discussions. At the end of each education presentation, the audience was encouraged to ask questions, and discussion ensued. These sessions were conducted with men and women present.

**Figure 2:**
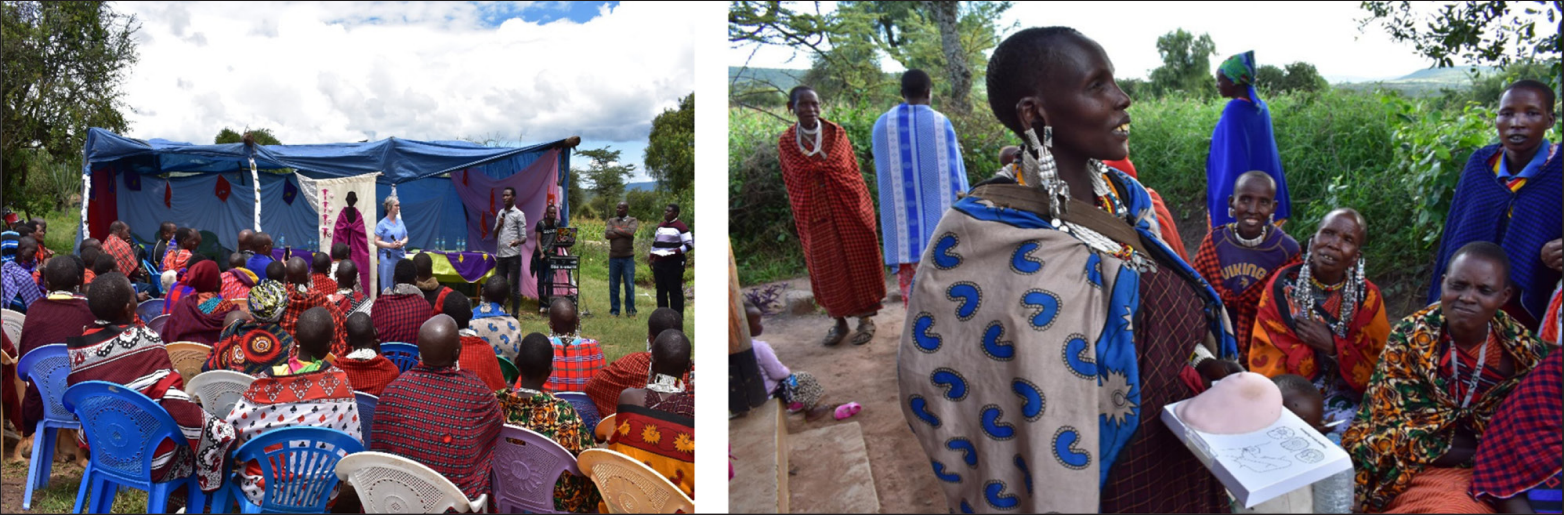
**Interactive nature of the educational presentations.** At the marketplace presentations, participants were encouraged to engage with the educational materials and ask questions.

A total of 32 presentations were given over the course of six weeks in the spring of 2018. Educational programs were performed at clinics, locations close to clinics (marketplaces), or rurally with a concurrently-established mobile clinic. District health officers advertised clinics in weeks prior at these respective sites, encouraging all women to attend. If they were performed at a clinic, they began at the start of each CACX screening clinic session and were repeated periodically throughout the day as newcomers arrived. If the presentation was held at the marketplace, it was established one week prior to the date of the CACX screening day at the local clinic. Men were encouraged to listen and to engage with the presenters as well (Figure 2). The same sessions were also presented at the two district hospitals for an audience of healthcare workers (HCWs) not involved in the screening efforts. Content addressed in the presentation included:

- Describing the problem of CACX
- Reviewing the structure and function of the cervix as it relates to other female reproductive organs
- Discussing prevention versus cure and the different ways to prevent CACX such as with the HPV vaccine and screening
- Explaining the concept of “screening” and preventative health
- Emphasizing the importance of visiting the hospital for CACX screening when feeling well, because of the lengthy asymptomatic period
- Demystifying the pelvic exam using simulation teaching aids
- Discussing cryotherapy as a treatment option for early-stage CACX
- Answering questions from the audience

**Figure 3:**
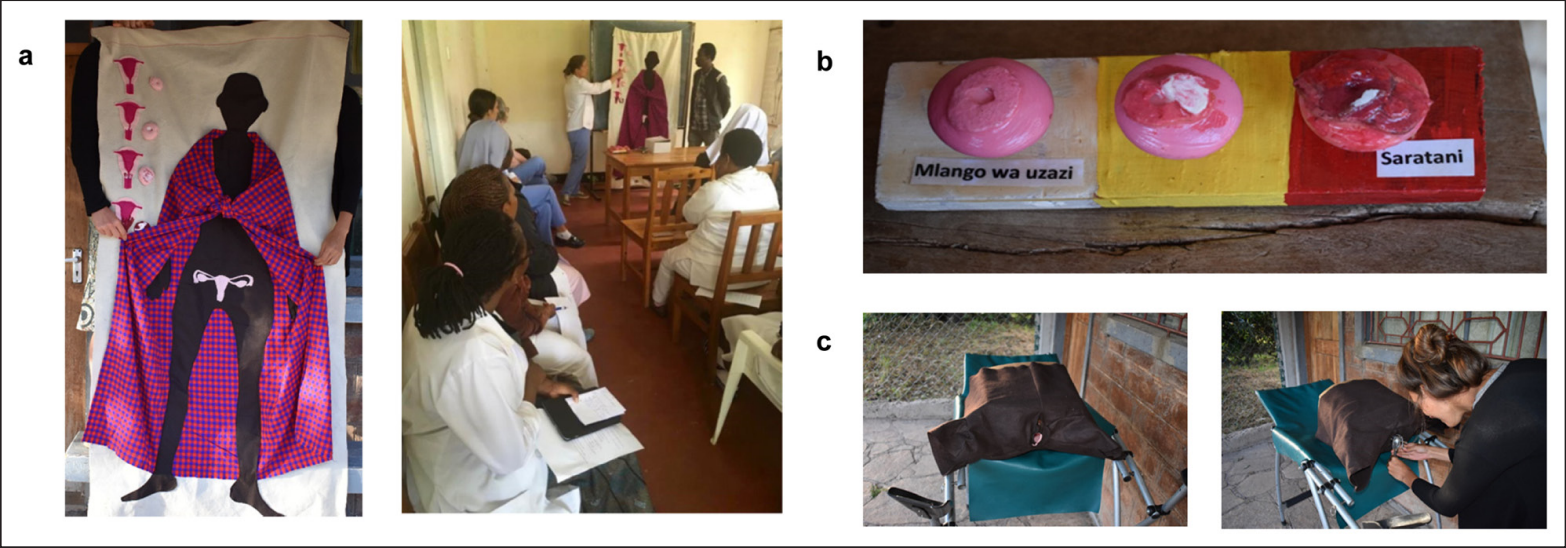
**Depicting anatomy, pathophysiology, and the pelvic exam in a culturally sensitive manner. a.** Anatomy was shown in situ superimposed on a human figure to help orient the women as we found that isolated pictures of organs in the human body which are “hidden” in the body did not register with the women. The uterus and cervix were discussed in terms of pregnancy and delivery as this is a familiar process to these women. **b.** The healthy cervix was shown alongside treatable pre-cancer and invasive cancer. The women held props made from doorknobs with painted clay “lesions,” which represented the progression from normal to invasive cervical cancer. **c.** A pelvic model made from felt and a cardboard box facilitated demonstration of the speculum exam. Women were also allowed to hold the speculum in order to demystify this tool.

The three-dimensional models were designed to be culturally appropriate, with the same skin tone and dress of their population. Early in the partnership, it was apparent from audience questions that depictions of reproductive organs isolated from the body did not register with women, as these organs are “hidden.” It was difficult for women to conceptualize that the cervix was connected the uterus and the vagina when seen in anatomical drawings from textbooks. In response, the anatomy was displayed in situ, superimposed on a human figure to help orient women (Figure 3A). A felt model represented a life-sized female body silhouette, and a superimposed uterus, fallopian tubes, and ovaries indicated approximate anatomical position. The figure was enrobed in a shuka cloth, a traditional garment worn by Maasai women. The same poster also displayed a felt uterus in coronal cross-sections and the cervix in full view. Fabric paint was applied to these sections to demonstrate the progression of cervical cancer from healthy to pre-cancerous lesions to growth beyond the reproductive tract. The entire model was made from felt and was easily folded up at the end of the clinic day to be transported to the next site.

Wooden models of the progression of cervical cancer were constructed using doorknobs with felt, paint, and lacquer overlay. Three cervical replicas were mounted on a wooden board to demonstrate the progression of cervical cancer from a localized to systemic disease (Figure 3B). The models were passed around during the education presentation. The texture of the models (smooth normal “cervix” versus rough and lumpy abnormal “cervix”) was emphasized in order to demonstrate the nature of cancer and progression of disease.

Many women in the audience had never experienced a pelvic exam. As this can be an anxiety-provoking procedure, the process of a pelvic exam was demonstrated using a three-dimensional pelvic model. The pelvic model was designed with felt supported by a cardboard backing, allowing for the insertion of a speculum for demonstration purposes. The model featured internal structures, including a vagina. Externally, the model featured a vulva, labia, and a urethra. The intent was to accurately represent the local population, and as female circumcision is widely practiced in the Maasai community, the model did not emphasize the labia majora. Insertion of the speculum was demonstrated during the education presentation, and women could hold the speculum in order to demystify this tool (Figure 3C).

At each clinic site, an instructional poster was displayed next to the gynecological examining table for the patient to see how to safely position herself, as the use of a gynecologic table was unfamiliar to most participants. A local woman was depicted in the poster laying her kanga, or wrap, on the table as is the local custom. Written KiSwahili instructions and phonetic translations were available for HCWs to describe the instructions in Maa language.

The script for the program was written in English language initially. Blinded back-translation was used to ensure fidelity of information. To make the presentation culturally sensitive, translation needed to be adapted from the word-for-word version. Furthermore, there were inherent linguistic challenges with the process of translating into Maa language as, traditionally, the language does not have a written form. To overcome this, a locally based, trilingual interpreter helped to culturally translate concepts such as “cancer,” which do not have an equivalent word in Maa. Cultural translation refers to a method of translation that replaces words in English with KiSwahili or Maa phrases that are culturally relevant for the target culture [16]. To validate that the message was not lost in translation, a trilingual interpreter was filmed performing the presentation in KiSwahili, and then again in Maa. The videos were replayed and interpreted back into English by separate interpreters. In addition, a local midwife was consulted to revise the script, incorporating language that is commonly used during gynecological examinations in the area. For example, the uterus and cervix were discussed in the context of pregnancy and delivery of the fetus, as this is a familiar process to these women.

**Figure 4:**
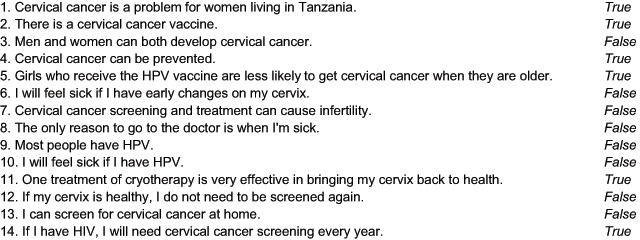
**Educational Screening Quiz.** The statements were designed after observations of local beliefs and utilization of the local health care clinic.

To evaluate the effectiveness of the patient education program, pre and post tests were administered before and after the education presentation. The quiz contained 14 true or false questions (Figure 4). Male and female HCWs at two district hospitals voluntarily participated in the pre and post quiz as well as groups of Maasai and non-Maasai, KiSwahili-speaking women. The latter groups were compensated for their time while the HCWs were not. Just as was done with the education program script, the quiz questions were developed in English and then translated into KiSwahili and Maa verbally. All quizzes were administered to the women by study staff. The exact translation for these study participants taking the pre and post surveys was in part translator-dependent. Descriptive statistical analysis was used for the survey data. Paired t-tests were used to compare survey scores pre and post attending the educational sessions.

Attendance at screening clinics and percentage of women receiving cervical cancer screening care was analyzed after completion of the educational program in 2018. Indications for screening were based on guidelines recommended by Pathfinder International. Screening with VIA was offered to women 30–49 years old and not menopausal. HIV-positive and HIV-negative women were deemed eligible to be tested with VIA [17]. Women who received cryotherapy were provided with return precautions and a prescription for doxycycline, as well as ibuprofen and sanitary pads. Patients were provided with a booklet to track screening dates, results, and recommended repeat screening. Women from previous years returned for a one-year follow-up if they had received cryotherapy. Local midwifes were also available to follow up with women if cryotherapy was performed after a positive screen. The clinic also offered clinical breast exams and HIV screening to all women regardless of age. Women were taught how to perform self-breast exams and become acquainted with their own breast tissue. Rapid HIV testing was also performed per Tanzanian Ministry of Health standards, in which local health care workers performed and recorded HIV testing and offered counselling to patients. Women were referred to a tertiary hospital if something was found to be abnormal on clinical exam, and local health officers were notified to allow for adequate follow-up for referred patients.

## Results

Table 1 presents baseline perceptions among the three different groups that were administered quizzes. Before the program intervention, the greatest discrepancy in test scores among groups pertained to the statement “The only reason to go to the doctor is when I’m very sick.” 5.8% of Maasai women responded “False” to this, with over 50% of the non-Maasai and HCWs responding “False” (Table 1). Additionally, healthcare workers were unanimous before the intervention with the statement “cervical cancer can be prevented.”

**Table 1:** Baseline Perceptions, % Correct.

Statement	Maasai (*n* = 17)	Non-Maasai (*n* = 15)	HCWs (*n* = 34)

1. Cervical cancer is a problem for women living in Tanzania.	76.5	93.3	85.3
2. There is a cervical cancer vaccine.	41.2	80	94.1
3. Men and women can both develop cervical cancer.	29.4	33.3	67.6
4. Cervical cancer can be prevented.	35.3	86.7	100
5. Girls who receive the HPV vaccine are less likely to get cervical cancer when they are older.	23.5	53.3	82.4
6. I will feel sick if I have early changes on my cervix.	76.5	40	50
7. Cervical cancer screening and treatment can cause infertility.	64.7	53.3	76.5
8. The only reason to go to the doctor is when I’m sick.	5.8	53.3	55.9
9. Most people have HPV.	5.8	66.7	47.1
10. I will feel sick if I have HPV.	41.2	26.7	32.4
11. One treatment of cryotherapy is very effective in bringing my cervix back to health.	29.4	93.3	35.3
12. If my cervix is healthy, I do not need to be screened again.	70.6	46.7	85.3
13. I can screen for cervical cancer at home.	94.1	86.7	82.4
14. If I have HIV, I will need cervical cancer screening every year.	35.3	66.7	67.6

HCWs = health care workers.

Results from the quiz administered before and after the program showed improvement of knowledge in all groups (Table 2). Maasai participants demonstrated a significantly greater (21.4%, p < 0.001) improvement in knowledge following the educational session, compared to the non-Maasai women and HCWs. Non-Maasai women also demonstrated moderate, significant improvement. Unsurprisingly, healthcare workers had the highest pre-test score with moderate, significant improvement after the intervention.

**Table 2:** After the educational intervention, a significant improvement in quiz scores was seen in all groups.

	*n*	Pre Score Mean (%)	Post Score Mean (%)	Improvement (%)	*P* value

Maasai	17	44.3	65.7	21.4	<0.001
Non-Maasai	15	61.9	73.3	11.4	<0.05
Health Care Workers	34	68.9	84.9	16	<0.001

Paired T test analyses demonstrated a significant improvement in quiz scores after the educational intervention among all groups. Score mean indicates average percentage scored correct.

**Figure 5:**
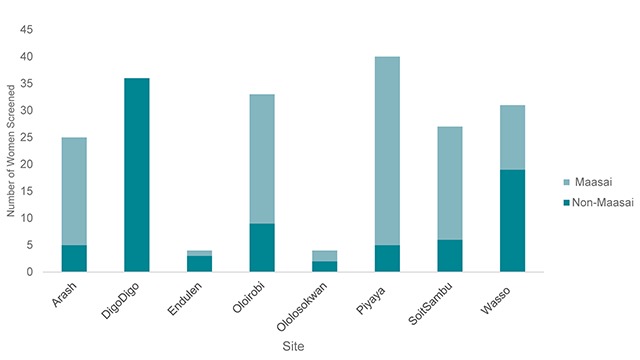
Distribution of Maasai Screened for CACX Post-Intervention Varied by Site.

Screening data after the educational intervention is presented in Table 3. 200 women were screened for CACX in the months the educational program took place. Screening attendance varied by region, with the greatest number of women presenting to the Piyaya clinic. The overall percentage of Maasai women screened increased from 40% before the intervention to 58% after the intervention. Of note, the percentage of Maasai women receiving CACX screening differed by region across the eight clinical sites (Figure 5). No women presenting to the DigoDigo clinic for CACX screening were Maasai, whereas the majority of women screened at Arash, Oloirobi, Piyaya and SoitSambu identified as Maasai. No trends were appreciated by distinguishing location by the setting of the educational intervention (Supplemental Table 1).

**Table 3:** Characteristics of Sample Screened for CACX, Post-Intervention.

Characteristics	Value

Participants, *n*	200
Ethnicity, *n (%)*	
Maasai	115 (58)
Non-Maasai	85 (42)
Mean age (SD)	37.6 (2.7)
Site, *n (%)*	
Arash	25 (12.5)
DigoDigo	36 (18.0)
Endulen	4 (2.0)
Oloirobi	33 (16.5)
Olosokwan	4 (2.0)
Piyaya	40 (20.0)
SoitSambu	27 (13.5)
Wasso	31 (15.5)

## Discussion

The culturally sensitive education program was successful in improving patient understanding of CACX and screening services. There was significant improvement in quiz scores post-intervention, by all groups, and the most so with the Maasai women. Through use of visual aids, in situ representation of relevant anatomy, and culturally appropriate translation into Maa, the language barrier to promoting health literacy was seemingly overcome. These results are similar to the findings of a study in which a unique data collection strategy, incorporating a visual pictogram, was used to overcome the barrier of illiteracy [18].

The improvement in the quiz scores could also be influenced by close collaboration with community members. As the UVM LCOM team worked with the interpreter and local HCWs to translate the patient education material linguistically and culturally, there was an ongoing dialogue, and much was learned about how different cultures perceive information and respond to questions. Adjustments were made to the education program in an iterative fashion. Additionally, the team had equal representation from UVM LCOM and local Tanzanians which potentially helped to show patients that the foreign team was locally accepted. Through this partnership, and the willingness of the UVM LCOM team to incorporate feedback from local people at the end of the presentations, the education program was enriched.

When working with healthcare interpreters in the US, the expectation is a word-for-word interpretation; however, this method may not be adequate for truly foreign concepts. In KiSwahili and Maa, there is no word for “cancer,” and many of the anatomical names do not directly translate. Having a native Maasai person, who was also familiar with Western culture as a trilingual interpreter, helped to bring a nuanced interpretation to the education program. Using culturally appropriate interpretation preserved the cultural context, which may be lost in word-for-word interpretation at the cost of the patient’s comprehension.

Common themes were identified by the research team from the discussions that ensued at the end of the presentations. Women voiced that they liked having information presented in visuals and conveyed they felt the effect to be more impactful. Women especially appreciated the helpfulness of the pelvic model and speculum, as many were learning about cervical cancer for the first time and had never seen a speculum before. However, there was ambivalence regarding the pelvic exam. Women expressed concerns about the cold temperature of the speculum, associating cold with a negative connotation. Additionally, there were fears that the speculum could cause infertility or injury to the bowel, even after the anatomy was discussed with accompanying visual depiction during the educational sessions. There were also general fears of the discomfort of objects being inserted into the vagina.

Anecdotally, the pelvic exam is thought to be a significant barrier in choosing to be screened in this population. The use of self-collected HPV tests could eliminate the need for pelvic exams all together. Such methods are used successfully in other low-resource settings, such as Appalachia and urban Peru. The HPV test is the most sensitive and specific test for predicting cervical cancer risk. HPV testing is integrated into World Health Organization algorithms for screening and treatment in low-resource settings. However, the infrastructure required for laboratory equipment can be costly.

When asked if they would be amenable to self-swabbing, one woman said, “I do not want to put anything in my vagina.” Fear of a speculum exam proved the largest barrier to choosing to be screened. Additionally, the belief that women didn’t need to be screened because they were asymptomatic was an ongoing barrier despite education. One woman commented, “I saw a person die of cervical cancer, but I’m still too scared to get screened.”

Finally, in assessing the impact of the screening program, we compared data that assessed Maasai use of CACX screening services before and after the educational intervention. 40% of women attending the pilot CACX clinic in 2017 were Maasai, and after the educational intervention, 58% of women attending the clinics were Maasai. However, attendance of Maasai women was not proportionally represented among all regions, and there could be confounding factors affecting our interpretation of increased attendance being secondary to the educational intervention. In the district of Wasso, the clinic charged a fee to patients who wished to access screening services in 2017. This was changed in 2018, the year the educational intervention was implemented, so this intervention could partially account for an increase in Maasai women accessing services. In the village of Endulen, an elephant was roaming the forest between the village and hospital on the dates the mobile clinic was set up, making travel by foot to the hospital dangerous, possibly accounting for the small number of patients screened. The difference in attendance in different regions may also speak to the heterogeneity of the Maasai culture. We are likely just scraping the surface in our understanding of this concept. Future work is needed to assess the impact of our educational intervention in the setting of Maasai representation in CACX screening.

This educational initiative could serve as a model for global health interventions in low-resource settings. Specifically, utilizing culturally-tailored visuals seemed to resonate with the Maasai women after hearing their perspectives at the end of the educational presentations. In addition, improving patient education improves the informed consent process for these women by promoting patient understanding. Informed consent is often overlooked in this area, where doctors are highly regarded, and so busy they seem unapproachable. Though the Maasai women showed the greatest improvement in post-test scores, they still had the overall lowest scores. Given this, there should be a continued specific focus on the Maasai women’s experience with CACX and access to healthcare to ensure representative participation of CACX clinics.

## Conclusions

Overall, the integration of culturally sensitive patient education in a marginalized population showed benefit. Knowledge of CACX at the screening clinics increased in the target population. Maasai representation among screening participants increased from 40 to 58% after the educational intervention was implemented. Such an education program could be adapted to fit other populations and address health concerns to promote cultural sensitivity.

## Additional File

The additional file for this article can be found as follows:

10.5334/aogh.2503.s1 Supplemental Table 1. 2018 Distribution of Attendance Post-Intervention.
