# Supplementary material for: Development and Implementation of a Culturally Appropriate Education Program to Increase Cervical Cancer Screening among Maasai Women in Rural Tanzania
Source: Ann Glob Health. 2019 Oct 17;85(1):127. doi: 10.5334/aogh.2503 (PMC6798900; doi:10.5334/aogh.2503)
Supplement: Supplemental Table 1. — 2018 Distribution of Attendance Post-Intervention. [file agh-85-1-2503-s1.pdf]

***Supplemental Table 1*. 2018 Distribution of Attendance Post-Intervention**

**Village**	**Total women screened for CACX**	***n* Maasai (%)**	**Site for Educational Program**
SoitSambu	27	21 (78)	Market
Ololosokwan	4	2 (50)	Clinic
Arash	25	20 (80)	Community meeting
Piyaya	40	35 (88)	Market
Oloirobi	33	24 (73)	Clinic
Endulen	4	1 (25)	Clinic
DigoDigo	36	0 (0)	Market
Wasso	31	12 (39)	Market
